# Computer Modeling Explains the Structural Reasons for the Difference in Reactivity of Amine Transaminases Regarding Prochiral Methylketones

**DOI:** 10.3390/ijms23020777

**Published:** 2022-01-11

**Authors:** Iris S. Teixeira, André B. Farias, Bruno A. C. Horta, Humberto M. S. Milagre, Rodrigo O. M. A. de Souza, Uwe T. Bornscheuer, Cintia D. F. Milagre

**Affiliations:** 1 Institute of Chemistry, UNESP-São Paulo State University, Araraquara 14800-060, SP, Brazil; iris.teixeira@unesp.br (I.S.T.); humberto.milagre@unesp.br (H.M.S.M.); 2 Institute of Chemistry, Federal University of Rio de Janeiro, Rio de Janeiro 21941-909, RJ, Brazil; bfarias.andre@gmail.com (A.B.F.); bruno.horta@gmail.com (B.A.C.H.); rodrigosouza@iq.ufrj.br (R.O.M.A.d.S.); 3 Department of Biotechnology & Enzyme Catalysis, Institute of Biochemistry, Greifswald University, 17487 Greifswald, Germany

**Keywords:** amine transaminase, chiral amines, biocatalysis

## Abstract

Amine transaminases (ATAs) are pyridoxal-5′-phosphate (PLP)-dependent enzymes that catalyze the transfer of an amino group from an amino donor to an aldehyde and/or ketone. In the past decade, the enzymatic reductive amination of prochiral ketones catalyzed by ATAs has attracted the attention of researchers, and more traditional chemical routes were replaced by enzymatic ones in industrial manufacturing. In the present work, the influence of the presence of an α,β-unsaturated system in a methylketone model substrate was investigated, using a set of five wild-type ATAs, the (*R*)-selective from *Aspergillus terreus* (Atr-TA) and *Mycobacterium vanbaalenii* (Mva-TA), the (*S*)-selective from *Chromobacterium violaceum* (Cvi-TA), *Ruegeria pomeroyi* (Rpo-TA), *V. fluvialis* (Vfl-TA) and an engineered variant of *V. fluvialis* (ATA-256 from Codexis). The high conversion rate (80 to 99%) and optical purity (78 to 99% *ee*) of both (*R*)- and (*S*)-ATAs for the substrate 1-phenyl-3-butanone, using isopropylamine (IPA) as an amino donor, were observed. However, the double bond in the α,β-position of 4-phenylbut-3-en-2-one dramatically reduced wild-type ATA reactivity, leading to conversions of <10% (without affecting the enantioselectivity). In contrast, the commercially engineered *V. fluvialis* variant, ATA-256, still enabled an 87% conversion, yielding a corresponding amine with >99% *ee*. Computational docking simulations showed the differences in orientation and intermolecular interactions in the active sites, providing insights to rationalize the observed experimental results.

## 1. Introduction

Optically pure amines and their derivatives play an important role in the chemical, agrochemical, and pharmaceutical industries, being key building blocks for the synthesis of pharmaceuticals and pesticides [1,2,3,4]. Their synthesis through classical approaches generally employs transition metal catalysis, which should be avoided, if possible, due to the scarcity of the metal resources and their toxicity [5]. An alternative for metal-catalysis is biocatalysis, using enzymes for chemical transformations [6]. This approach is advantageous since enzymes generally act under mild reaction conditions, typically display high chemo-, regio- and stereoselectivity, originate from renewable resources, and are biocompatible, biodegradable, and rarely present toxicity [5,7,8].

Among the biocatalytic approaches to synthesizing optically pure amines, one very attractive option is the use of amine transaminases (ATAs). These enzymes are pyridoxal-5′-phosphate (PLP)-dependent enzymes and belong to the PLP-fold classes I and IV [9]. They catalyze the transfer of an amino group from an amine donor to an aldehyde or ketone and can be (*R*)- or (*S*)-selective [10,11,12]. The reaction can be classified as a neutral redox reaction, consisting of two half-reactions: the first step is an oxidative deamination of the amino donor, converting the PLP9—bound in the active site of the enzyme—to its reduced form, PMP, followed by the reductive amination of the amino acceptor, when the PMP is again oxidized to PLP [11,12]. As the cofactor is regenerated, it is required only in catalytic amounts [13].

Amine transaminases can be employed in the synthesis of chiral amines through the kinetic resolution of a racemic mixture of amines, asymmetric synthesis using prochiral ketones, or via the deracemization of racemic amines [14]. Herein, we focused on the asymmetric synthesis of chiral amines, which can achieve a theoretical yield of 100%. One major drawback of this methodology is the thermodynamically disadvantageous equilibrium of the reaction [15]. For ideal asymmetric synthesis, amino donors and acceptors must be more reactive than their respective co-products, thus making the reaction equilibrium more favorable. In addition, the use of an excess of an amino donor is perhaps the easiest way to shift the equilibrium toward product formation [12,13,14]. In the last few decades, a number of academic and industrial examples using ATAs for chiral amine production have been reported in the literature [16,17,18,19,20,21,22,23].

Nevertheless, there is no general rule for substrate acceptance by the transaminases and, sometimes, even small differences in the chemical structure of the substrate, such as the presence or absence of an α,β-unsaturation adjacent to the carbonyl function, can lead to different enzymatic reactivities [24]. Therefore, better comprehension of the relationship of substrate structure and enzyme interaction is helpful and necessary when planning synthetic routes. 

In this paper, we aim to contribute to the understanding of the substrate scope of some (*R*)- and (*S*)-selective wild-type ATAs, and an (*S*)-selective engineered amine transaminase variant toward the asymmetric synthesis of chiral amines, using a conformationally restricted α,β-unsaturated model ketone and its saturated analog, via experimental and docking approaches.

## 2. Results and Discussion

Initially, 4-phenylbutan-2-one (1a) was employed as a model substrate and isopropylamine (IPA) was chosen as an amino donor since moderate yields for this transformation have been already reported [24]. Different (*S*)- and (*R*)-selective enzymes were chosen and analyzed for conversion and enantiomeric excess (Table 1).

The transamination reaction, using **1a** as a substrate mediated by *Chromobacterium violaceum* ATA, led to good conversions and excellent selectivity (Entry 1, Table 1). A similar approach has been reported by Kroutil and co-workers, using L-Ala as an amino donor, achieving poor to good conversions (16% and 86%) depending on the recycling system, respectively, LDH/GDH and AlaDH/FDH, without affecting the enantiomeric excess (~50% *ee*) [25]. Reports of the *R. pomeroyi* ATA reaction in the presence of IPA afforded the desired product in moderate yields (67 to 75%), as shown by Bornscheuer and co-workers [26] but, under the conditions studied herein, complete conversion and enantiomeric excess of > 99% *ee* could be achieved (Entry 2, Table 1).

The (*R*)-selective transaminase from *Aspergillus terreus* (Entry 3, Table 1) has also been investigated by several groups, always delivering very good selectivity (> 99% *ee*) for the desired transformation but with poor conversion results when IPA was used as the amino donor (46%), as reported by Strohmeier and co-workers [27]. Another (*R*)-selective ATA from *Mycobacterium vanbaaleni* was also evaluated (Entry 4, Table 1) and, to the best of our knowledge, there are no previous reports on the reaction between **1a** and IPA as an amino donor. The work of Bornscheuer and co-workers is the only one to evaluate its reactivity when D-Ala was used as the amino donor, with an LDH/GDH recycling system [28]. It is worth mentioning that the use of (*R*)-selective transaminases is far less prevalent than using the (*S*)-selective ones and it is of the utmost importance to identify novel (*R*)-selective transaminase to produce optically pure enantio-complementary amine compounds [29,30].

The *V. fluvialis* transaminase has also been used with moderate results in terms of conversion and selectivity but only when using L-Ala as the amino donor; in our case, the use of IPA as the amino donor led to the desired chiral amine with good conversions and selectivity (Entry 5, Table 1) [31,32,33]. The ATA-256 from Codexis is an engineered variant of *V. fluvialis* that has five mutations [34]. Results in terms of conversion, when using IPA as the amino donor and compound **1a** with ATA-256, were the same compared to the *V. fluvialis* wild-type enzyme; however, enantioselectivity with the engineered enzyme was significantly higher (Entry 6, Table 1). To the best of our knowledge, there are no data in the literature regarding the use of ATA-256 to convert **1a**. 

Taking a closer look at the literature, searching for similar substrates to **1a**, it is interesting to note that most substrates have saturated alkyl chains; the only exception is the planar 4-phenylbut-3-yn-2-one, for which good results have been reported using the (*S*)-selective *Chromobacterium violaceum* transaminase [35]. The α,β-unsaturated ketone 4-phenylbut-3-en-2-one (**2a**) has been studied by Gotor-Fernandez and co-workers, where moderate to good conversions and optical purity could be obtained only with commercially available Codexis enzymes, such as the (*R*)-selective ATA-024, ATA-033 and ATA-415 and the (*S*)-selective ATA-254, ATA-256 and ATA-260 [36]. The fact that the reaction of the α,β-unsaturated ketone (*E*)-4-phenylbut-3-en-2-one (**2a**) as a substrate and IPA as an amino donor, catalyzed by *Chromobacterium violaceum*, *Arthrobacter citreus,* and *Arthrobacter* sp. wild-type transaminases, resulted in conversions of <1%, and that some genetically modified (*R*)- and (*S*)-selective transaminases resulted in conversions ranging from 24 to 57% and an optical purity varying from 84 to >99% *ee*, has drawn our attention [36,37,38]. Thus, we decided to evaluate the ability of the abovementioned enzymes concerning the transaminase reaction between (*E*)-4-phenylbut-3-en-2-one (**2a**) and IPA as an amino donor regarding the synthesis of this unsaturated chiral amine (Table 2).

The first experiments, where *C. violaceum*, *R. pomeroyi*, *A. terreus*, *M. vanbaalenii* and *V. fluvialis* were used, reminded us of the unsatisfactory results for the desired substrate when using wild-type transaminases. Conversions were poor, although they did have excellent selectivity. As reported by Gotor-Fernandez and co-workers, the ATA-256 from Codexis was the best transaminase capable of converting 4-phenylbut-3-en-2-one (**2a**) into the desired chiral amine (**2b**) with a reasonable conversion rate [36]. The difference in reactivity of this α,β-unsaturated ketone has drawn our attention, to try to understand what the differences in the active site of the ATAs could be that could drive the reaction in the direction of chiral amine synthesis.

**Figure 1:**
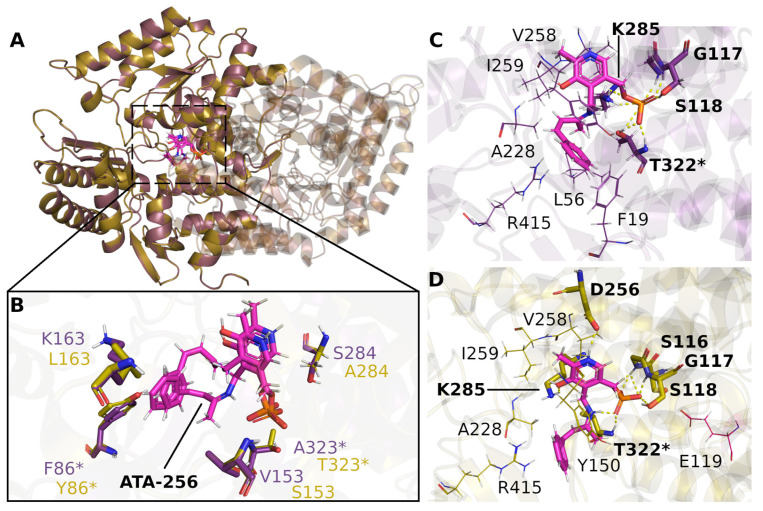
Comparison of the poses obtained by the molecular docking of **2a** in the transaminase from *V. fluvialis* (purple) and ATA-256 (yellow). (**A**) Unconserved amino acids within a 5 Å radius of compound **2a** are highlighted (**B**). Binding poses of **2a** with *V. fluvialis* (**C**) and ATA-256 (**D**) transaminases, as suggested by docking studies. Hydrogen bonds are shown by yellow dashed lines and amino acids marked with * show that they belong to the B chain.

Modeling studies were carried out, with a two-fold objective: first, to evaluate possible structural differences favoring the conversion of **2a** (Figure 1B) when using wild-type (*S*)- and (*R*)-selective transaminases; second, to characterize and compare the ligand-protein interactions of **2a,** with a homology model of ATA-256 and its respective template from *V. fluvialis* (PDB code 4E3Q) (Figure 1C,D).

Molecular docking runs were performed to study the binding mode of the considered compounds in the active site of the transaminases. Figure 1 shows a comparison of binding modes of compound **2a** in *V. fluvialis* and ATA-256; both are (*S*)-selective enzymes. It is important to note that ATA-256 has five amino acid mutations, leading to significant changes in the binding site (Figure 1B). The hydrophobic amino acids, V153 and A323, from *V. fluvialis* are replaced by polar amino acids in ATA-256 (S153 and T323); charged and polar amino acids (K163 and S284) are replaced by hydrophobic residues (L163 and A284). We hypothesize that these changes in amino acid properties are the main ones responsible for the different recognition by ATA-256. Compound **2a** seems to bind to ATA-256 in an orientation that favors the reaction, with a relatively small distance to the nitrogen atom of K285 (2.5 Å). For the wild-type protein, this distance is about 4.0 Å.

**Figure 2:**
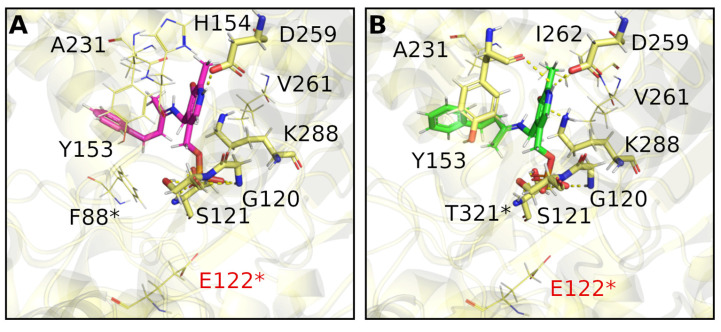
Binding poses of compounds **2a** (**A**) and **1a** (**B**) in the binding site of the *C. violaceum* ATA obtained by molecular docking. Hydrogen bonds are shown by yellow dashed lines. Amino acids marked with * show that they belong to the B chain; those colored red represent unfavorable contacts.

Furthermore, the interactions between this compound and the amino acids of the active site of ATA-256 seem to be more complementary, compared to the wild-type interactions. More specifically, **2a** can make more hydrogen bonds and Van der Waals interactions with ATA-256 compared to the wild-type form of *V. fluvialis* (Figure 1B,C and Supplementary Table S3). With the Cvi-ATA, substrate **2a** showed no conversion to the respective amine, whereas its structural analog **1a** resulted in an 80% conversion. This difference in the conversion of **2a** in relation to **1a** may indicate that the presence of the double bond had a negative influence on the activity of this enzyme. The best pose of molecular docking showed that compound **2a** had fewer hydrogen-bond interactions with the binding site compared to compound **1a** (Figure 2A and Supplementary Table S4), probably due to the decrease in flexibility of the molecule promoted by the double bond.

**Figure 3:**
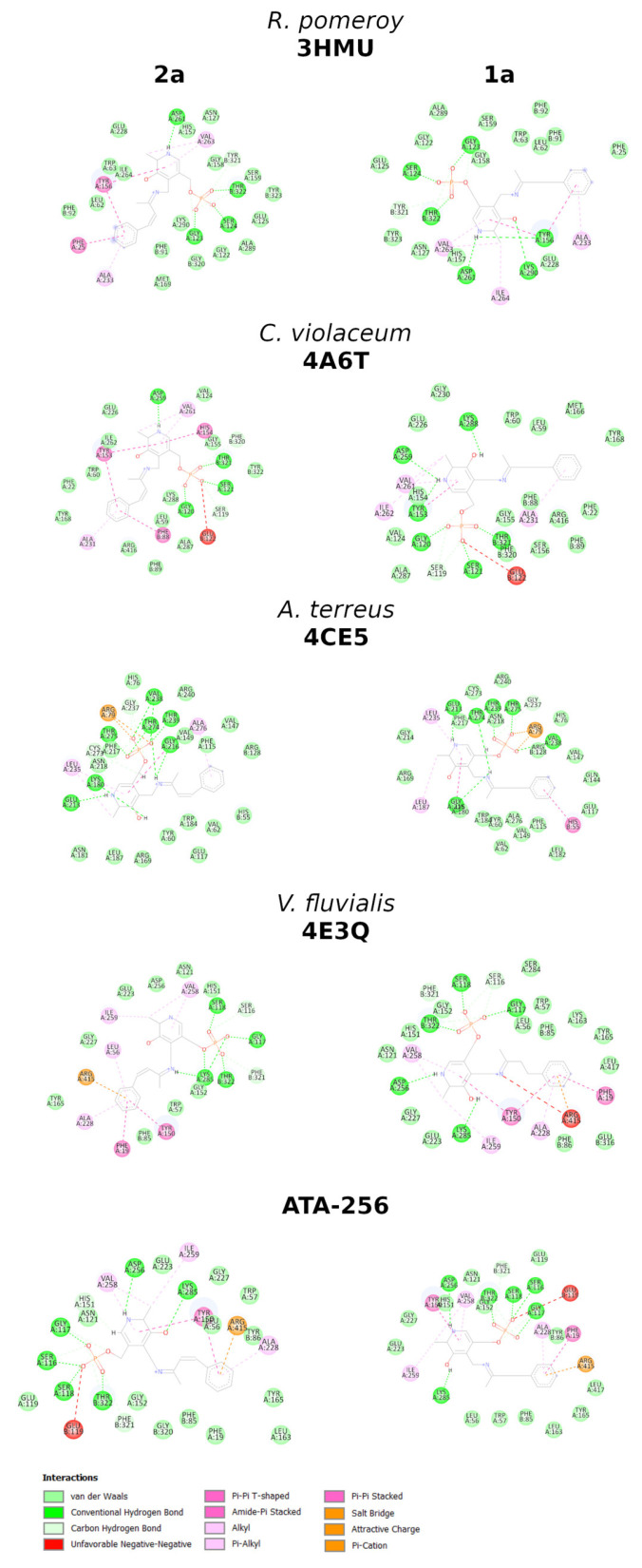
Analysis of the interactions obtained by the molecular docking of **2a** and **1a**.

We performed in silico studies to identify positive or negative interactions between the substrate and amino acids around the active site of the (*R*)-selective *Atr*-ATA, aiming to rationalize the observed results. Molecular docking studies showed that, despite the fact that external aldimine intermediate with substrates **1a** and **2a** can be correctly oriented in the active site at an appropriate distance to suffer a nucleophilic attack, small differences observed in the binding mode were able to justify the different substrate conversion rates. As well as in the case of (*R*)-selective Cvi-ATA, we can observe the effect of the double bond in decreasing the substrate conversion due to the decreased flexibility of compound **2a** compared to **1a**. Figure 3 shows that K180 is interacting via hydrogen bonding with the carbonyl in compound **2a** and, probably, this interaction should interfere with the lysine attack on the substrate, since it was not observed in compound **1a**. Besides that, compound **1a** is able to interact via π-π stacking with H55*, this not being observed for compound **2a**.

## 3. Materials and Methods

### 3.1. Materials

The ketones 4-phenylbutan-2-one and (*E*)-4-phenylbut-3-en-2-one, titanium (IV) isopropoxide, ammonia solution 2.0 M in ethanol, pyridoxal 5′-phosphate hydrate, and isopropylamine are commercially available from Sigma–Aldrich^®^, Saint Louis, MO, USA. 

Nuclear Magnetic Resonance (NMR) spectra were recorded on a Bruker Fourier 300 (B0 7.1 T) spectrometer, at operating frequencies of 75 MHz for ^13^C NMR- and 300 MHz for ^1^H NMR-spectroscopy. The chemical shifts (δ) are reported in parts per million (ppm), using, as an internal reference, tetramethylsilane (TMS, δ = 0.0) and, in the scale relative to CDCl_3_, 7.24 ppm for ^1^H NMR and 77.23 for ^13^C NMR. The coupling constants (J) were measured in Hertz (Hz) and were characterized as doublet (d), double doublet (dd), multiplet (m), singlet (s), broad singlet (bs), sextet (st), and triplet (t).

Infrared (IR) spectra were recorded on an FTIR spectrophotometer (Vertex 70—Bruker), equipped with attenuated total reflectance (ATR), operating at 400–4000 cm^−1^.

### 3.2. Analytical Methods

For purification by dry silica chromatography (DSC), silica 230–400 mesh from Macherey-Nagel was used. For purification by preparative thin-layer chromatography, Kieselgel DF silica from Riedel-deHaën at 0.75 mm, on glass plates of 20 cm × 20 cm, was used.

Analyses by gas chromatography (GC) for measuring the conversion and enantiomeric excess were performed using a Shimadzu GC-2010 Plus chromatograph, equipped with an autosampler AOC-20i and flame ionization detector (FID), and an Agilent 7890B gas chromatograph, coupled with a mass spectrometer, model 5977A. The column used for determining conversion was a Restek Rtx^®^-5 (30 m × 0.25 mm × 0.25 μm, diphenyl dimethyl polysiloxane) for the GC-FID, or a HP-5ms (30 m × 0.25 mm × 0.25 μm, (5%-Phenyl)-methylpolysiloxane) for the GC-MS. Enantiomeric excess was determined on a chiral column Hydrodex^®^ β-3P (25 m × 0.25 mm × 0.25 μm; Macherey-Nagel, Darmstadt, Germany).

Method 1 (RTX-5) parameters: injector: 260 °C; detector: 300 °C; constant flow 1.22 mL/min; 80 °C/hold 3 min, 80–300 °C/rate 30 °C/min; 280 °C hold 3 min.

Method 2 (Hydrodex **1b**) parameters: injector: 180 °C; detector: 180 °C; constant flow 0.9 mL/min; 155–175 °C/rate 1 °C/min, 175–185 °C/rate 10 °C/min, 180 °C/hold 10 min.

Method 3 (Hydrodex **2b**) parameters: injector: 180 °C; detector: 180 °C; constant flow 0.9 mL/min; 170 °C/hold 5 min, 170–180 °C/rate 5 °C/min, 180 °C/hold 10 min.

### 3.3. Expression and Production of Cell-Free Extracts Containing ATAs

*E. coli* BL21(DE3) pLysS cells were used for the recombinant expression of the proteins. The plasmids containing synthetic genes encoding amine transaminases from *Aspergillus terreus*, *Mycobacterium vanbaalenii*, *Ruegeria pomeroyi*, *Vibrio fluvialis,* and *Chromobacterium violaceum* were constructed in pET and pGASTON vectors. 

For the transformation, approximately 50 ng of the plasmids were added to 100 μL of competent cells. The cells were kept on ice for 30 min, followed by 2 min at 42 °C, then were incubated again on ice for 2 min. After that, 1 mL of SOC medium was added to the cells and they were incubated at 37 °C for 1 h, under orbital stirring (300 rpm). The growing cells were inoculated on LB (lysogenic broth) medium plates, supplemented with kanamycin or ampicillin, depending on the resistance antibiotic, and the plates were kept at 37 °C overnight.

For protein expression, 30 mL of an overnight culture was inoculated in 600 mL of LB medium, supplemented with the resistance antibiotic. Firstly, the culture was incubated at 37 °C and 130 rpm until an OD600 of 0.7 was achieved. Then, IPTG was added (final concentration 0.1 mM) for *C. violaceum*, *V. fluvialis* and *R. pomeroyi* ATAs, or rhamnose (0.2% *w/v*) for *A. terreus* and *M. vanbaalenii* ATAs. The time and temperature of induction varied, depending on the enzyme expressed. The induction occurred overnight for all the enzymes, with the exception of the *R. pomeroyi* ATA, where induction took 6 h. The temperature was set to 20 °C (*A. terreus* and *M. vanbaalenii*) or 30 °C (*C. violaceum*, *V. fluvialis* and *R. pomeroyi*). After expression, the cells were harvested by centrifugation (10 min, 15,000× *g*, 20 °C). 

The cell pellets were resuspended in cold lysis buffer (sodium phosphate buffer, pH 7.0, 20 mM, 20 μM PLP, 2 mM EDTA, 1 mM PMSF, 5% glycerol). They were then disrupted by sonication (Bandelin Sonopuls HD-2070), with 30–40% of the maximum power, in 9 cycles of 30 s, in continuous mode, with rest breaks of 1 min between cycles. All the procedures were conducted in an ice bath. The cell suspension was centrifuged (20 min, 20,000× *g*, 4 °C) to remove cell debris. The supernatant was pre-purified by dialysis, in cellulose tubes (pores of 14,000 Da), for 24 h at 4 °C. For every 8 mL of cell-free extract, 250 mL of dialysis buffer was used (sodium phosphate buffer, pH 7.5, 50 mM, 20 μM PLP). At the end of the process, aliquots were prepared, containing 1.0 mL of enzyme extract and 0.5 mL of 20% glycerol, and the cell-free extracts were stored in the freezer at −20 °C.

### 3.4. Bradford Assay

The Bradford reagent was made using 25 mg of Coomassie brilliant blue G-250, dissolved in 25 mL 95% ethanol [39]. To this solution were added 50 mL of 85% H_3_PO_4_. The resulting solution was added to 425 mL of distilled water and stored in a refrigerator.

For the analytical curve (see Supplementary Figure S1), a solution of 1 mg/mL of bovine serum albumin (BSA) was prepared, which was successively diluted to 0.5, 0.125, 0.062, 0.031, and 0.015 mg/mL. Samples were made by taking 20 μL of the BSA solution, 1.58 mL of Milli-Q water, and 0.4 mL of Bradford reagent, which were incubated at room temperature for 10 min. After the incubation period, the absorbance was measured at 595 nm. Analyses were performed in triplicate. For the negative control, 1.6 mL of Milli-Q water and 0.4 mL of Bradford reagent were mixed. For quantification of the enzymes, 20 μL of the crude cell lysate (post-dialysis) was used. The concentrations of the total proteins in crude cell lysates were: *C. violaceum* (0.426 mg mL^−1^), *A. terreus* (not quantified, below the range of the analytical curve), *V. fluvialis* (0.345 mg mL^−1^), *M. vanbaalenii* (0.033 mg mL^−1^), *R. pomeroyi* (0.247 mg mL^−1^).

### 3.5. General Procedure for Reactions with the Crude Cell Lysate

Into a 2 mL Eppendorf tube, 20 mM of the ketone was added, solubilized in 15 μL of DMSO (1% vv^−1^), followed by the addition of 1 mM of PLP and 300 mM of isopropylamine (prepared in sodium phosphate buffer, 100 mM, pH 7.5). After that, the crude cell lysate (1.5 mL) was added, and the pH was corrected to 7. The reaction was kept under orbital stirring (850 rpm) at 30 °C for 24 h. The reaction was quenched by the addition of NaOH (10 M) until a pH of 10–12 was reached, followed by extraction with ethyl acetate (2 × 1.5 mL). The organic phases were combined, then dried with anhydrous magnesium sulfate, and the solvent was evaporated.

### 3.6. General Procedure for Reactions with ATA-256

Into a 1.5 mL Eppendorf tube, 10 mM of the ketone was added, solubilized in 25 μL of DMSO (2.5% vv^−1^), followed by the addition of 1 mM of PLP and 1 M of isopropylamine (prepared in sodium phosphate buffer, 100 mM, pH 7.5). After that, the pH was corrected to 7, then the enzyme was added with the remaining buffer (total volume of 1 mL). The reaction was kept under orbital stirring (850 rpm) at 45 °C for 24 h. The reaction was quenched by the addition of NaOH (10 M) until a pH of 10–12 was reached, followed by extraction with ethyl acetate (2 × 1.5 mL). The organic phases were combined, then dried with anhydrous magnesium sulfate, and the solvent was evaporated.

### 3.7. Reductive Amination for the Synthesis of the Racemic Amines

Then, under an N_2_ atmosphere, to 0.5 mmol of the ketone substrate, 1 mmol of titanium isopropoxide (0.3 mL) and 2.5 mmol (1.25 mL) of ammonia in ethanol 2 M were added [40]. The reaction system was stirred at room temperature in an inert atmosphere for 6 h. Then, 0.75 mmol (28 mg) sodium borohydride was added and the reaction system was kept under stirring for 3 h. The reaction was quenched by the addition of 2 mL of ammonium hydroxide 2 M. The inorganic precipitate was filtered under reduced pressure and was abundantly washed with ethyl acetate. The aqueous phase was extracted with ethyl acetate (2 × 5 mL) and the organic phases were combined, washed with brine, dried with anhydrous magnesium sulfate, and the solvent was evaporated. See supporting information for NMR spectra and GC-FID and HPLC chromatograms. 

To summarize: 4-phenyl-2-butanamine (**1b**). 99% yield. CAS Nr. 22374-89-6. MS *m/z* (%): 149 (3), 132 (65), 117 (44), 103 (12), 91 (100),77 (26). IR (ATR) (cm^−1^): 3412, 3331, 3027, 2924, 1561, 1451, 1362, 688.^1^H NMR- (300 MHz, CDCl_3_) δ 1.12 (d, *J* = 6.3 Hz, 3H), 1.68 (m, 4H), 2.78–2.55 (m, 2H), 2.93 (st, *J* = 6.3, 6.3, 6.3, 6.3, 6.3 Hz, 1H), 7.35–7.07 (m, 5H). ^13^C NMR (75 MHz, CDCl_3_) δ: 24.05, 33.00, 41.87, 46.78, 125.96, 128.54,128.57, 142.45. GC-FID (after acetylation): column—Hydrodex β−3P, method 2, tr = 21.623 (S) min and 22.066 (R) min. An absolute configuration was assigned, in comparison with Liu et al. [2]: (Chiralcel OD-H, i-propanol/hexane = 4/96, flow rate 0.5 mL/min, λ = 220 nm); tr = 37.92 min (*R*) and 45.73 min (*S*).

(*E*)-4-phenyl-3-buten-2-amine (**2b**). Purified by dry-silica flash chromatography (dichloromethane:methanol 10%), 66% yield. CAS Nr. 51616-91-2. MS- *m/z* (%):147 (69), 146 (49), 132 (100), 130 (25), 115 (63), 91 (24); IR-(cm^−1^): 3350, 3283, 2961, 1584, 969, 755, 680. ^1^H NMR- (300 MHz, CDCl_3_) δ 1.28 (d, *J* = 6.5 Hz, 3H), 2.13 (sl, 2H), 3.80–3.52 (m, 1H), 6.21 (dd, *J* = 15.9, 6.7 Hz, 1H), 6.48 (d, *J* = 15.9 Hz, 1H), 7.42–7.15 (m, 5H). ^13^C NMR- (75 MHz, CDCl_3_) δ: 23.75, 49.57, 126.50, 127.57, 128.54, 128.76, 135.56, 137.25. GC-FID (after acetylation): column—Hydrodex β−3P, method 1, tr = 26.307 min (*S*) and 26.936 min (*R*). Absolute configurations were assigned in comparison with Liu et al. [2], (HPLC Chiralcel AD-H, i-propanol/hexane = 10/90, flow rate 1.0 mL/min, λ = 254 nm); tr = 5.88 min (*R*) and 6.83 min (*S*). 

### 3.8. General Procedure for the Acetylation of Amines

To 1.0 mL of ethyl acetate, 1 mg of the amine, 10 μL acetic anhydride, and a small crystal of DMAP were added. The system was stirred for 10 min, and after that the organic phase was washed with NaOH (2 M), dried with anhydrous magnesium sulfate, and analyzed.

### 3.9. General Procedure for in Silico Studies

Molecular structures of ligands were prepared using Avogadro and the energy was minimized using the PM3 semi-empirical method available in the Gaussian 09 package. The 3D structures of the proteins *A. terreus* (PDB 4CE5 [41], 1.63 Å resolution), *C. violaceum* (PDB 4A67 [42], 1.80 Å resolution), *V. fluvialis* (PDB 4E3Q [43], 1.90 Å resolution) and *R. pomeroyi* (PDB 3HMU, 1.56 Å resolution) were taken from their respective crystal structures, then prepared by removing the water molecules and adding the missing atoms. The protonation states of the amino acids were assigned according to a pH value of 7. For ATA-256 [34], no crystal structure was available, and a 3D model was built using homology modeling. For this purpose, a search for suitable templates was carried out using BLAST on the NCBI server (see Supplementary Table S1). The chosen template was the aminotransferase from *Vibrio fluvialis* (PDB 4E3Q). The model was constructed using the SwissModel server [44]. The stereochemical quality of the model was assessed by the Ramachandran plot on the MolProbity server [45], and z-score on the Prosa-web server [46].

### 3.10. Molecular Docking 

The parameters for docking were determined by an analysis of poses obtained by redocking, where the quality criterion was the root-mean-square deviation (RMSD) value (see Supplementary Table S2). The docking of ligands in transaminase from *A. terreus* was performed via the ChemPLP scoring function, and the binding site radius was set to 20.0 Å around the nitrogen atom (ID2862) of Lys180. Twenty runs of the genetic algorithm were performed for each molecule. However, the same set of parameters did not perform well when docking the ligands in the cavity of the transaminases from *C. violaceum* and *R. pomeroyi*. In such cases, the Chemscore function and a radius of 10 Å around Lys288 (PDB 4A6T) and Lys290 (PDB 3HMU) were used. The optimal parameters for docking on *V. fluvialis* were the ChemPLP scoring function and a radius of 10 Å around the nitrogen (ID 4385) of Lys285. Figure 2 and Figure 3 were prepared using PyMOL version 2.4.0a0 [47] and the BIOVIA Discovery Studio Visualizer v.16.1.0.15350 [48]. The GOLD software v.2020.1 was used in the docking studies [49].

## 4. Conclusions

These molecular docking simulations shed light on experimental data and helped rationalize the generally lower conversion rates and optical purities found for the less flexible α,β-unsaturated ketone **2a,** compared to **1a**. The high conversions (80 to 99%) and enantioselectivities (78 to 99% *ee*) for **1a,** with IPA as an amino donor, were far superior to those observed for the α,β-unsaturated substrate **2a,** no matter whether the (*R*)- or (*S*)-selective wild-type ATAs or the engineered ATA-256 were used because there are more favorable intermolecular interactions in the active site, due to the higher flexibility of compound **1a** compared to **2a**.

Most importantly, the difference in conversions of **2a** with the (*S*)-selective wild-type transaminases from *V. fluvialis* compared to its engineered variant ATA-256 from Codexis could be rationalized. Molecular docking simulations suggested that the four mutations, V153S, K163L, S284A, and A323T, in the engineered ATA-256 variant are the main ones responsible for changes in the transaminase active site, resulting in more possibilities for intermolecular interactions and favorable distances, leading to observed differences in higher conversion rates and optical purities for **2a** when using the commercially engineered *V. fluvialis* variant ATA-256 (87% conversion, >99% *ee*), whereas the wild type afforded only a 5% conversion with 90% *ee*.

## Figures and Tables

**Table 1:** Conversions and enantiomeric excess (% *ee*) values of amine product **1b,** obtained by asymmetric synthesis using isopropylamine (IPA) as the donor and crude cell lysate from *Escherichia coli* containing the overexpressed ATA.

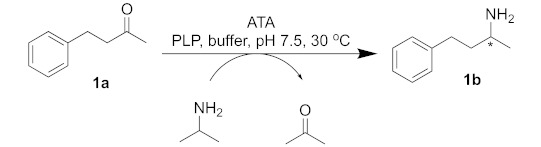			
Entry	ATA	Conv. (%)	(% *ee*)
1	*C. violaceum*	80	97 (*S*)
2	*R. pomeroyi*	>99	>99 (*S*)
3	*A. terreus*	93	>99 (*R*)
4	*M. vanbaalenii*	>99	>99 (*R*)
5	*V. fluvialis*	97	78 (*S*)
6	ATA-256	>99	>99 (*S*)

Conditions: 20 mM **1a**, 300 mM IPA, 1% DMSO, 1 mM PLP, 1.5 mL crude cell lysate, 30 °C, 24 h. Conversions were determined via gas chromatography (GC-FID). The enantiomeric excess was determined via chiral GC analysis using a Hydrodex^®^ β-3P column (25 m × 0.25 mm × 0.25 μm; Macherey-Nagel). The ‘*’ represents an asymmetric carbon which its absolute configuration could be R or S depending on the used enzyme.

**Table 2:** Conversions and enantiomeric excess (% *ee*-values) for the amine product **2b** obtained by asymmetric synthesis using isopropylamine (IPA) as a donor and crude cell lysate from *E. coli*, containing the overexpressed ATA.

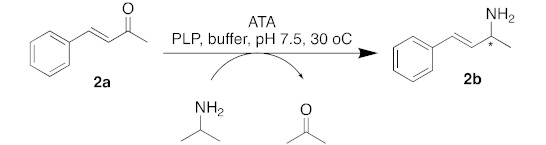			
Entry.	ATA	Conv. (%)	(% *ee*)
1	*C. violaceum*	0	-
2	*R. pomeroyi*	9	>99 (*S*)
3	*A. terreus*	3	>99 (*R*)
4	*M. vanbaalenii*	4	>99 (*R*)
5	*V. fluvialis*	5	90 (*S*)
6	ATA-256	87	>99 (*S*)

Conditions: 20 mM **2a**, 300 mM IPA, 1% DMSO, 1 mM PLP, 1.5 mL crude cell lysate, 30 °C, 24 h. Conversions were determined via gas chromatography (GC-FID). The enantiomeric excess was determined via chiral GC analysis using a Hydrodex^®^ β-3P column (25 m × 0.25 mm × 0.25 μm; Macherey-Nagel). * represents an asymmetric carbon which its absolute configuration could be R or S de-pending on the used enzyme.

## Data Availability

Data are included in the article.

## Supplementary Materials

The following supporting information can be downloaded at: https://www.mdpi.com/article/10.3390/ijms23020777/s1.
