# Supplementary material for: Computer Modeling Explains the Structural Reasons for the Difference in Reactivity of Amine Transaminases Regarding Prochiral Methylketones
Source: Int J Mol Sci. 2022 Jan 11;23(2):777. doi: 10.3390/ijms23020777 (PMC8776209; doi:10.3390/ijms23020777)

Computer modelling explains structural reasons for the difference in reactivity of amine transaminases towards prochiral methylketones

Iris S. Teixeira,[1] André B. Farias,[2] Bruno A. C. Horta,[2] Humberto M. S. Milagre,[1] Rodrigo O. M. A. Souza,[2] Uwe T. Bornscheuer,[3] Cintia D. F. Milagre[1]

[1] Institute of Chemistry, UNESP – São Paulo State University, Araraquara, SP, Brazil

[2] Institute of Chemistry, Federal University of Rio de Janeiro, Rio de Janeiro, RJ, Brazil

[3] Department of Biotechnology & Enzyme Catalysis, Institute of Biochemistry, Greifswald University, Greifswald, Germany

Supporting Information Data

Figure S1. Analytical curve – Bradford Assay

Figure S2. NMR Spectra ^{1}H (300.19 MHz, $CDCl_3$) of amine **1b**

Figure S3. NMR Spectra ^{13}C (75.48 MHz, $CDCl_3$) of amine **1b**

Figure S4. NMR Spectra ^{1}H (300.19 MHz, $CDCl_3$) of amine **2b**

Figure S5. NMR Spectra ^{1}H (75.48 MHz, $CDCl_3$) of amine **2b**

Table S1. The four best results for Blastp of ATA-256 sequence.

Table S2. Docking validation using redocking strategy with different scoring functions and radius around the reference atom. Rigid and flexible docking were performed.

Table S3: Comparison between interactions observed in docking studies of ATA-256 and *V. fluvialis* (4E3Q). Amino acids marked with * represent that they belong to B chain.

Table S4: Comparison between interactions observed in docking studies of *C. violaceum*. Amino acids marked with * represent that they belong to B chain.

Figure S6. Validation of model by z-score (A), local estimates based on the QMEAN scoring function (B) and ramachandran plot (C). Model of ATA-256 built by homology modeling (D) and the structural comparison with template 4E3Q (E).

Figure S7. Overview of protein structures and sequence of transaminase from *A. terreus* (A), *C. violaceum* (B), *R. pomeroyi* (C), *V. fluvialis* (D) and ATA-256 (E).

Figure S8. Analysis of interactions obtained by molecular docking of **2a** and **1a**.

Figure S1. Analytical curve – Bradford Assay

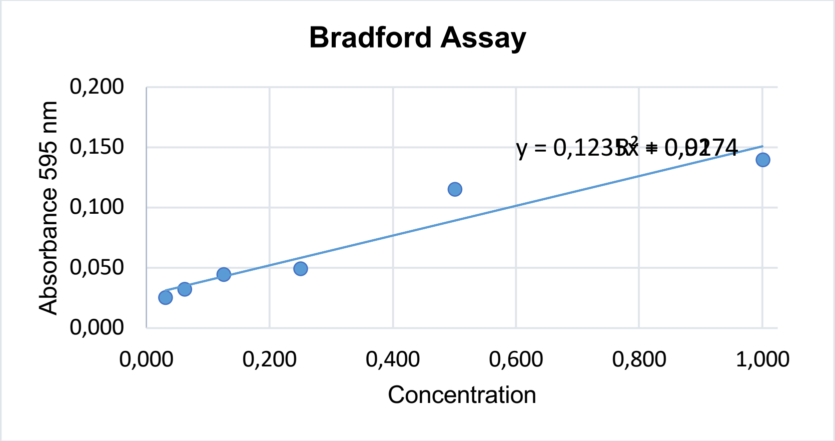

Figure S2. NMR Spectra ^{1}H (300.19 MHz, $CDCl_3$) of amine **1b**

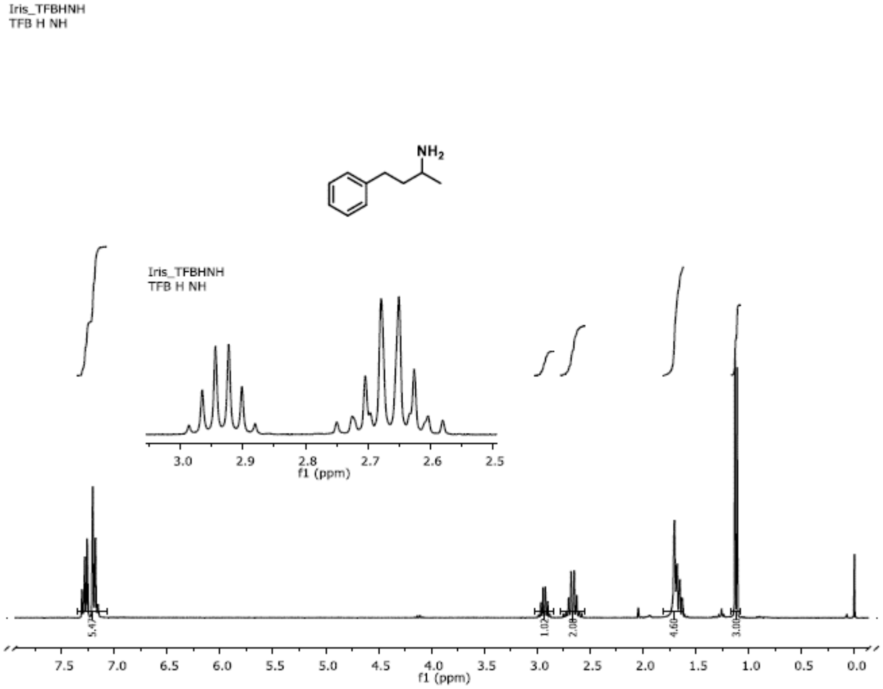

	Parameter	Value
1	Data File Name	C:/ Users/ Iris/ Google Drive/ Mestrado/ dados espectrometricos / RMN/ Iris_TFBHNH/ 10/ fid
2	Title	Iris_TFBHNH
3	Comment	TFB H NH
4	Origin	Bruker BioSpin GmbH
5	Owner	nmr
6	Site	
7	Spectrometer	FOURIER300
8	Author	
9	Solvent	CDCl3
10	Temperature	297.0
11	Pulse Sequence	zg30
12	Number of Scans	21
13	Receiver Gain	38
14	Relaxation Delay	1.0000
15	Pulse Width	11.3800
16	Acquisition Time	5.3687
17	Acquisition Date	2017-04-05T14:36:56
18	Modification Date	2017-04-05T14:39:04
19	Spectrometer Frequency	300.19
20	Spectral Width	6103.5
21	Lowest Frequency	-593.3
22	Nucleus	1H
23	Acquired Size	32768
24	Spectral Size	65536

Figure S3. NMR Spectra ^{13}C (75.48 MHz, $CDCl_3$) of amine **1b**

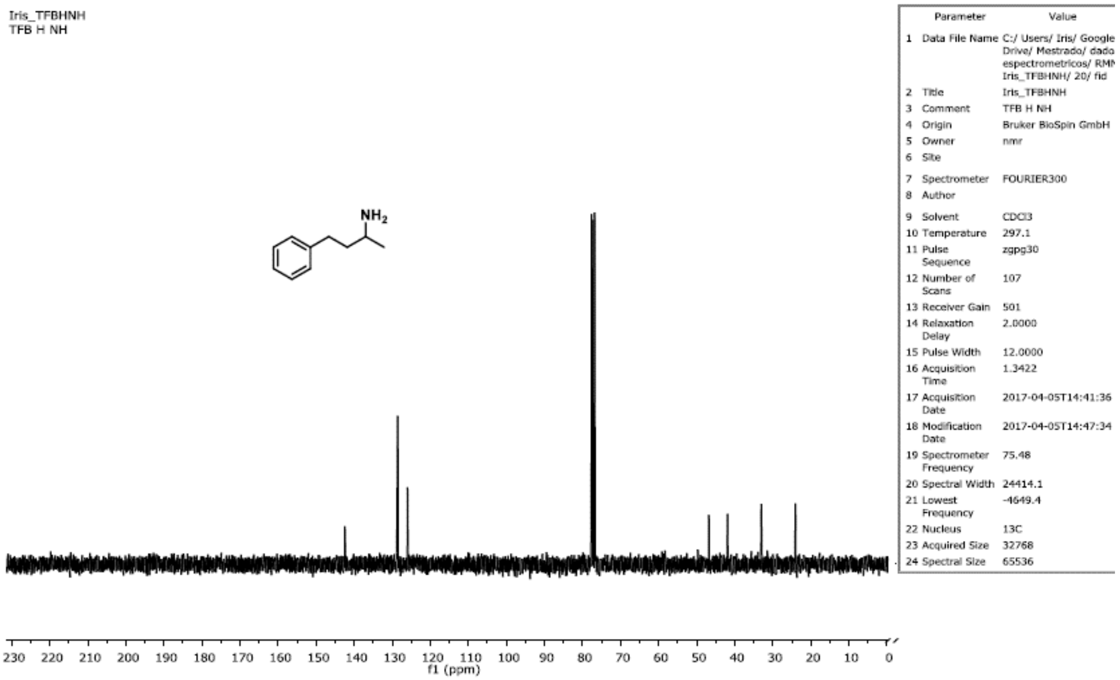

Figure S4. NMR Spectra ^{1}H (300.19 MHz, $CDCl_3$) of amine **2b**

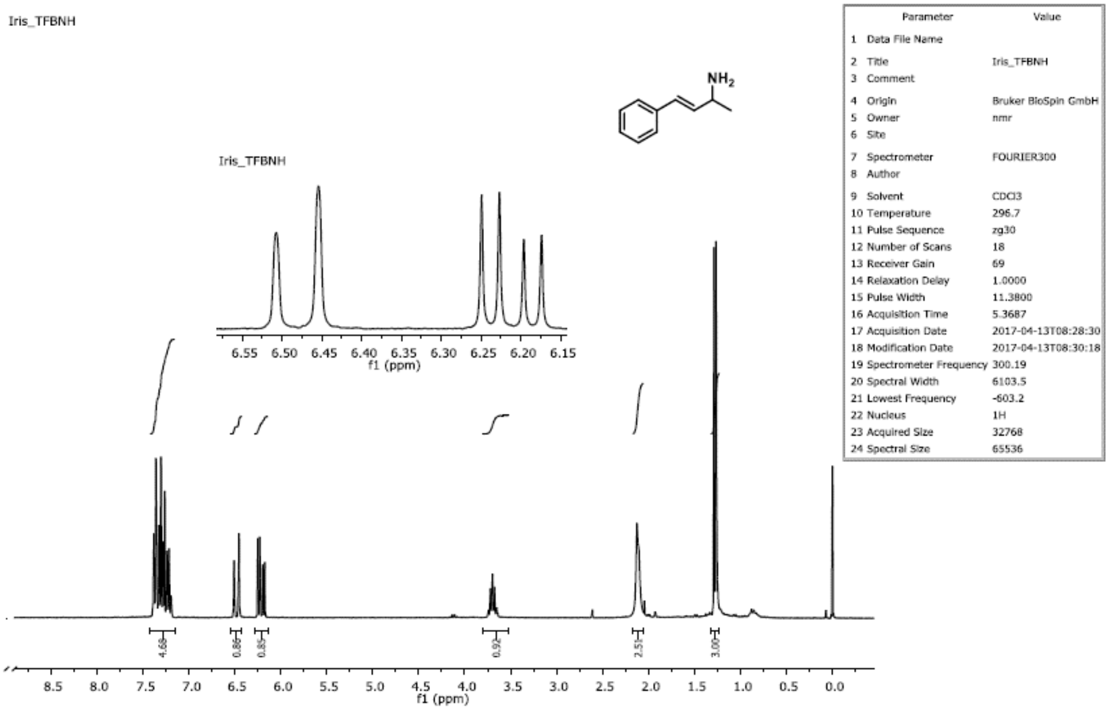

Figure S5. NMR Spectra ^{1}H (75.48 MHz, $CDCl_3$) of amine **2b**

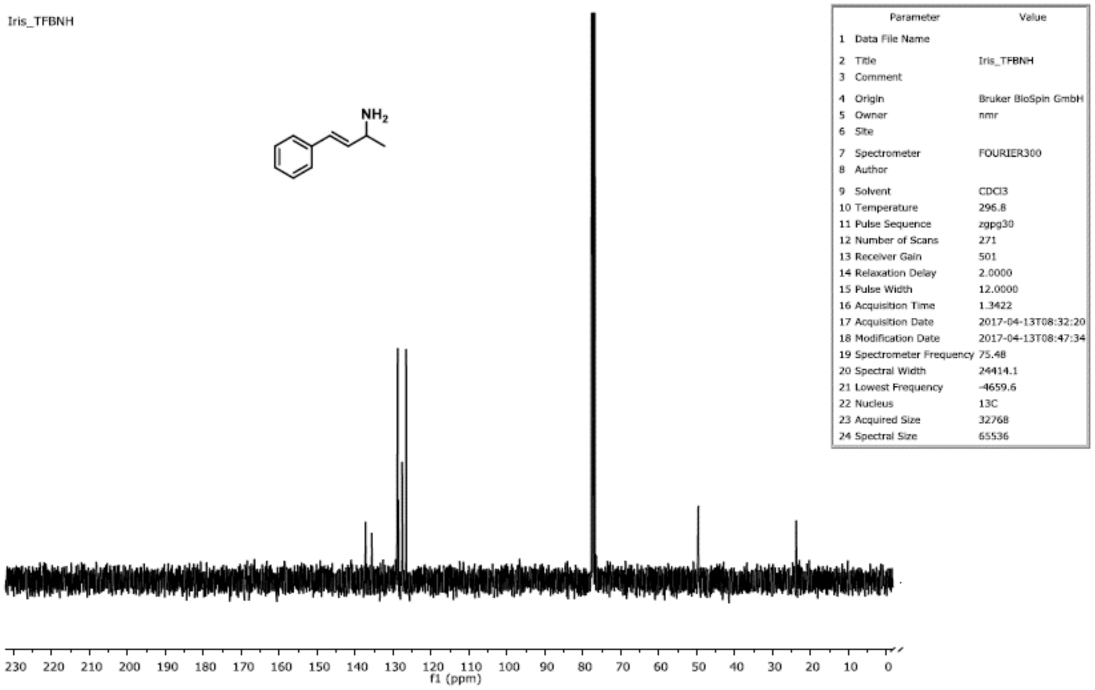

Table S1. The four best results for Blastp of ATA-256 sequence.

PDB	Cover (%)	Per. Identity (%)	Resolution (Å)	Organism
5ZTX	100	94.04	2.00	*V. fluvialis*
3NUI	100	94.04	2.00	*V. fluvialis*
4E3R	100	92.49	1.90	*V. fluvialis*
4E3Q	99.00	91.37	1.90	*V. fluvialis*

Table S2. Docking validation using redocking strategy with different scoring functions and radius around the reference atom. Rigid and flexible docking were performed.

PDB code	Function	Radius around ref.	RMSD
4CE5	Chemplp (rigid)	20	1.5321
	Chemscore (rigid)	20	0.9871
	Chemplp (flex)	20	1.6915
	Chemplp (flex)	10	1.7563
	Chemscore (flex)	20	1.3967
	Chemscore (flex)	10	1.5325
4E3Q	Chemplp (flex)	20	4.9960
	Chemplp (flex)	10	0.5673
	Chemscore (flex)	20	5.1090
	Chemscore (flex)	10	2.8099

Table S3: Comparison between interactions observed in docking studies of ATA-256 and *V. fluvialis* (4E3Q). Amino acids marked with * represent that they belong to B chain.

Type	ATA256		4E3Q	
	2a	1a	2a	1a
Hbond	116	116	-	-
	117	117	117	117
	118	118	118	118
	256	256	-	256
	285	285	285	285
	322*	322*	322*	322*
Van der Waals	15 residues	14 residues	11 residues	16 residues
Unfavorable	119*	119*	-	415
Pi-Pi Stacking	-	19	19	19
	150	150	150	150
Alkyl/Pi-Alkyl	-	-	56	-
	228	228	228	228
	258	258	258	258
	259	259	259	259
Pi-cation	415	415	415	415

Table S4: Comparison between interactions observed in docking studies of *C. violaceum*. Amino acids marked with * represent that they belong to B chain

Type	*C. violaceum*	
	2a	1a
Hbond	120	120
	121	121
	-	153
	259	259
	-	288
	321*	321*
Van der Waals	15 residues	17 residues
Unfavorable	122*	122*
Pi-Pi Stacking	153	153
	154	-
	88*	-
Alkyl/Pi-Alkyl	231	231
	261	261
	-	262

Figure S6. Validation of model by z-score (A), local estimates based on the QMEAN scoring function (B) and ramachandran plot (C). Model of ATA-256 built by homology modeling (D) and the structural comparison with template 4E3Q (E).

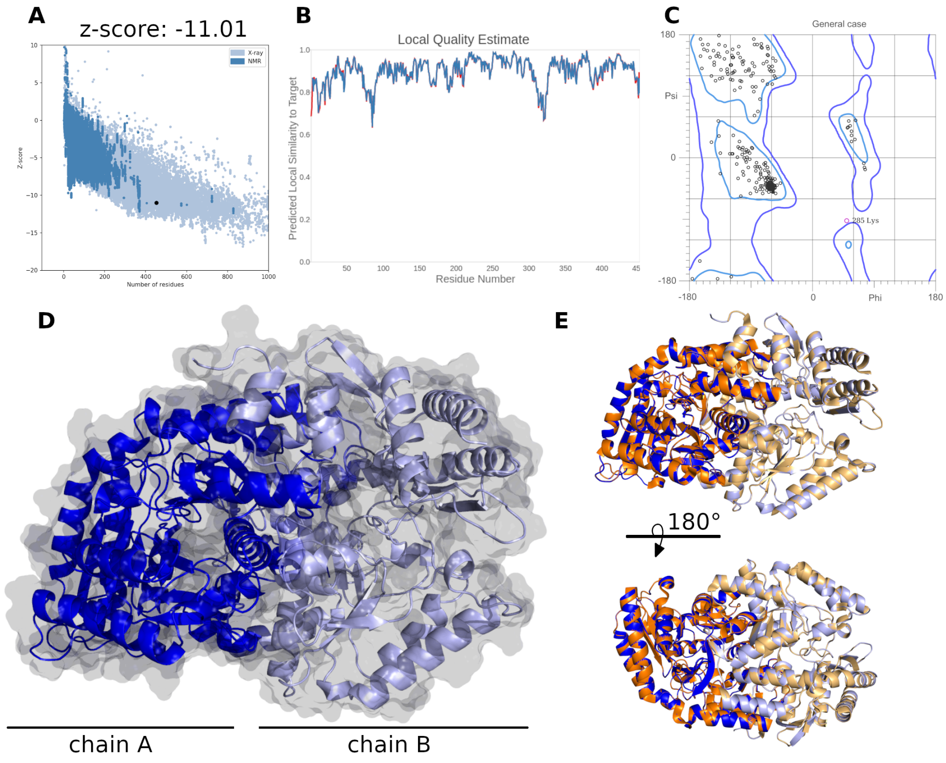

Figure S7. Overview of protein structures and sequences of the transaminase from *A. terreus* (A), *C. violaceum* (B), *R. pomeroyi* (C), *V. fluvialis* (D) and ATA-256 (E).

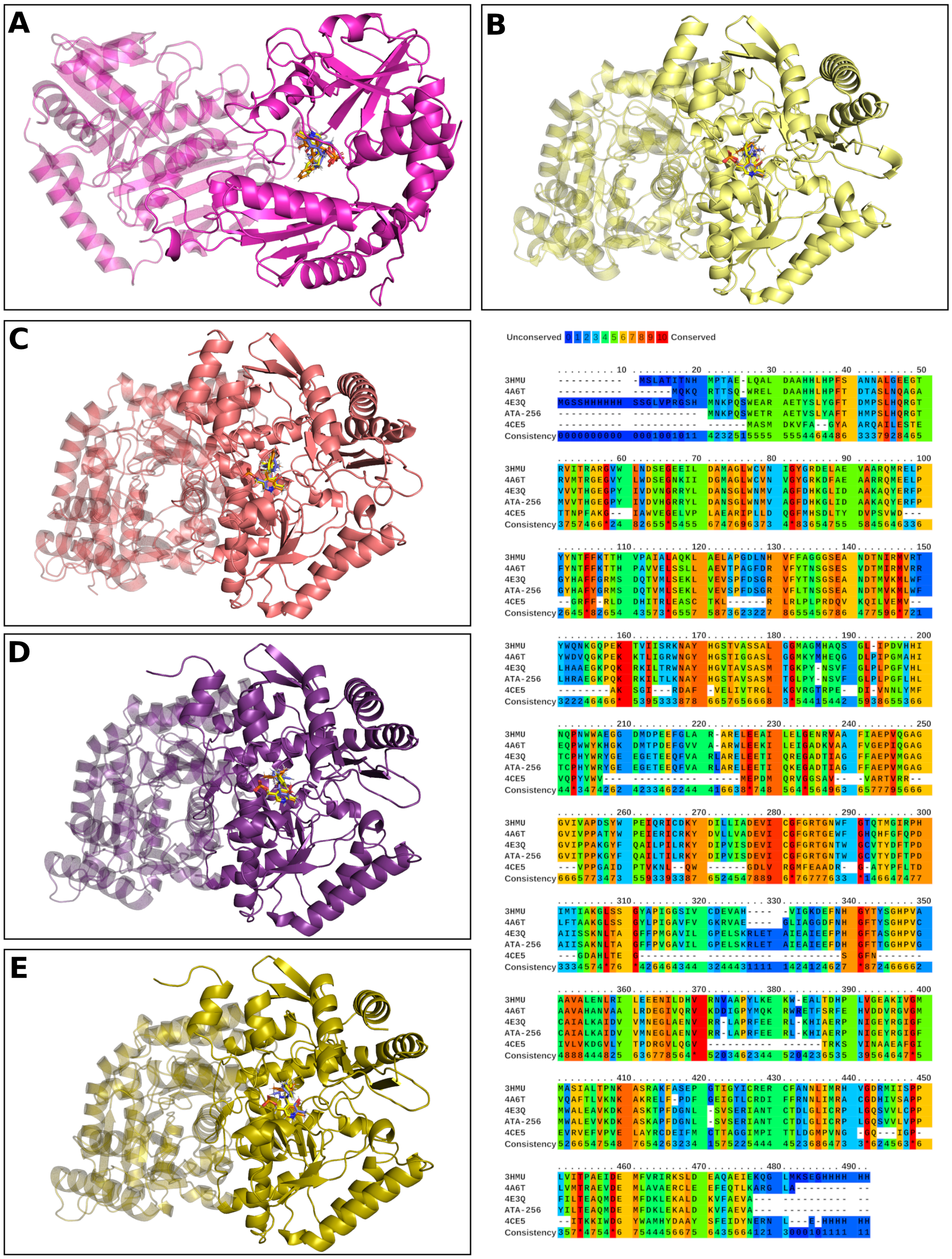

Figure S8. Analysis of interactions obtained by molecular docking of **2a** and **1a**.

R. pomeroy
3HMU

2a **1a**

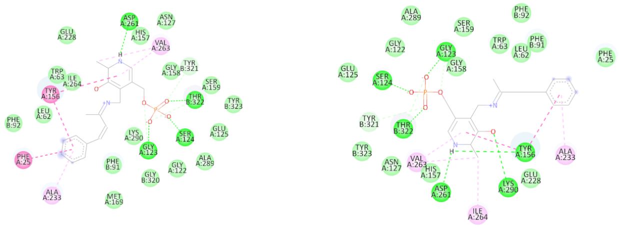

C. violaceum
4A6T

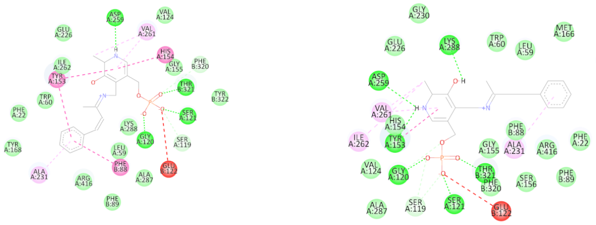

A. terreus
4CE5

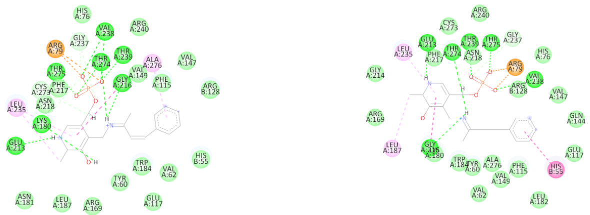

V. fluvialis
4E3Q

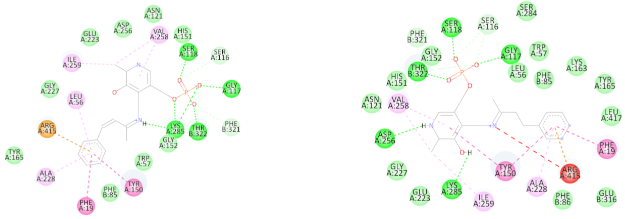

ATA-256

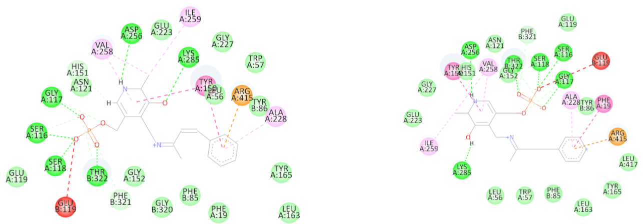

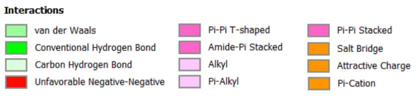

Supplement: Supplementary file 1 [file ijms-23-00777-s001.zip › ijms-1518982-supplementary.pdf]
